# Segmentation of Total Cell Area in Brightfield Microscopy Images

**DOI:** 10.3390/mps1040043

**Published:** 2018-11-19

**Authors:** Martin Čepa

**Affiliations:** Contipro a.s., Dolní Dobrouč 401, Dolní Dobrouč 561 02, Czech Republic; martin.cepa@contipro.com; Tel.: +420-467-070-316

**Keywords:** brightfield segmentation, microscopy, ImageJ, Fiji, image analysis, cells

## Abstract

Segmentation is one of the most important steps in microscopy image analysis. Unfortunately, most of the methods use fluorescence images for this task, which is not suitable for analysis that requires a knowledge of area occupied by cells and an experimental design that does not allow necessary labeling. In this protocol, we present a simple method, based on edge detection and morphological operations, that separates total area occupied by cells from the background using only brightfield channel image. The resulting segmented picture can be further used as a mask for fluorescence quantification and other analyses. The whole procedure is carried out in open source software Fiji.

## 1. Introduction

With the advances in fluorescence microscopy techniques and, especially, in the field of quantitative fluorescence detection, microscopes are becoming more and more tools of not just qualitative evaluations, but also quantitative measurements. This, however, also brings up the necessity of utilizing various methods of image analysis to obtain reliable results and the image segmentation, i.e., separation of areas in the image that are important for analysis from background, is one of the most crucial steps [1]. Probably the most common way of image segmentation is thresholding, that separates areas of interest from background using pixel values in fluorescence images, their spatial distribution, and other metrics [2,3,4,5,6,7]. This method, however, cannot be used in cases when the knowledge of the total area occupied by cells is necessary (for example for calculation of mean fluorescence intensity in cells, integrated fluorescence density etc.) and the labeling is localized in specific organelles and not in the whole cell volume. Another approach is the manual segmentation, that can be considered as a reference when done properly [8], but which is impossible to be used in high-throughput analysis.

In this protocol, we present a simple method that segments microscopy images using only brightfield channel. With modern fluorescence or confocal microscopes, it is not a problem to obtain a brightfield image simultaneously, or consequently, at a very fast pace together with fluorescence image, so the spatial localization of cells in both channels is the same. The areas that are recognized as cells in brightfield images can, thus, be easily applied as selections in fluorescence channels as well. There are methods of brightfield segmentation but, usually, they are not so simple and ready to use [9,10,11,12]. The proposed procedure is carried out using Fiji [13] and requires the installation of Canny edge detection plugin [14]; the rest of the functions are built-in. It does not require any additional equipment, and only a basic level of image analysis knowledge.

## 2. Experimental Design

The process is based on edge detection, which highlights areas of varying brightness across the image. This leads to the creation of a binary image with highly structuralized areas within areas occupied by cells. The binary image is further processed using maximum filter and basic morphological operations to fill the insides of cells, smooth the edges, and remove debris in the background. The resulting binary image clearly separates areas with strong edge contrast (i.e., cells) from background.

Knowledge of pixel resolution (in nm/pixel) in the analyzed image is required. If the segmentation is used for fluorescence analysis, all channels must have the same spatial resolution and, ideally, be acquired simultaneously. It is recommended not to include scale bars in analyzed images as it can be falsely positively detected. Otherwise, there are no other limitations; images in all channels should be acquired following basic rules considering the fluorescence crosstalk, saturation of the detector, spatial sampling frequency, etc. [15,16]

### Software

Fiji, based on ImageJ 1.51h (Wayne Rasband, National Institutes of Health, Bethesda, MD, USA)

## 3. Procedure

The presented procedure is carried out in Fiji software, purely because of its simple use and popularity. It can, however, be applied in any other image processing software if it supports the necessary functions (especially edge detection and morphological operations). Each step in the procedure includes a short explanation, ImageJ macro command, and illustrative pictures of processing brightfield images of HCT-116 cells The presented parameters are best suited for images acquired with high magnification objectives (40× and higher). Changes necessary for processing images acquired with lower magnification objectives are discussed in Section 4.

**Figure 1:**
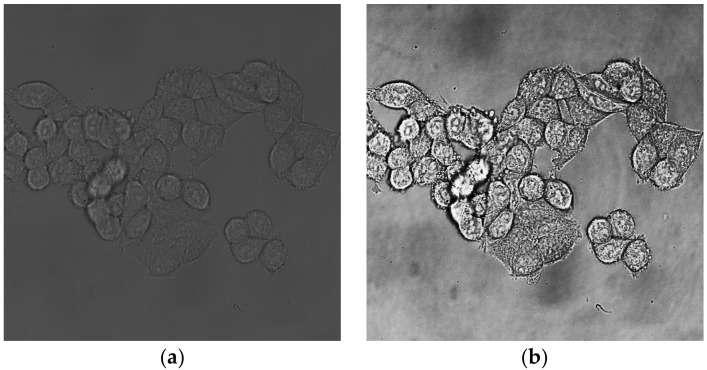
Brightfield channel of HCT-116 cell line. (**a**) Unedited picture; (**b**) picture with equalized histogram.

**Figure 2:**
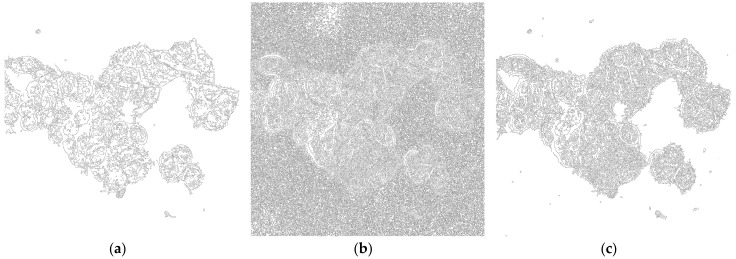
Edge detection using Canny algorithm on Figure 1b using Gaussian kernel radius of 1.75, low threshold 0.1, and varying values for High threshold. (**a**) High threshold set to 20; (**b**) High threshold set to 2.0; (**c**) High threshold set to 8.0.

**Figure 3:**
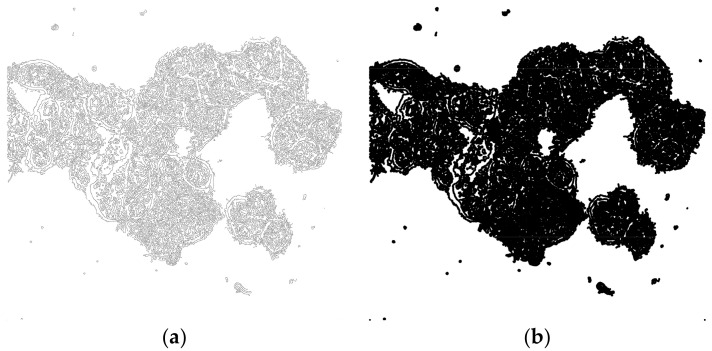
Effect of Maximum filter. (**a**) Edged imaged before filtration. (**b**) Image after filtration using radius = 5 pixels.

**Figure 4:**
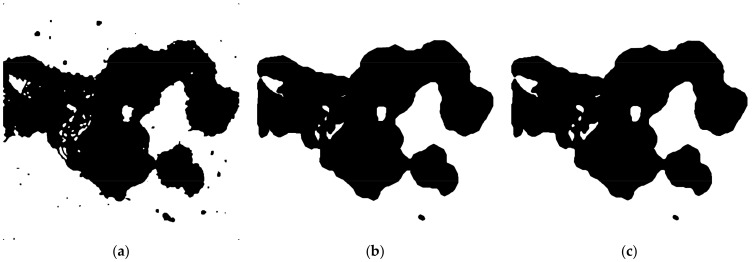
Processing of filtered image (Figure 3b) using morphological operations. (**a**) Closing for filling holes; (**b**) Opening for removing debris; (**c**) Erosion to shrink the volume of segmented area.

**Figure 5:**
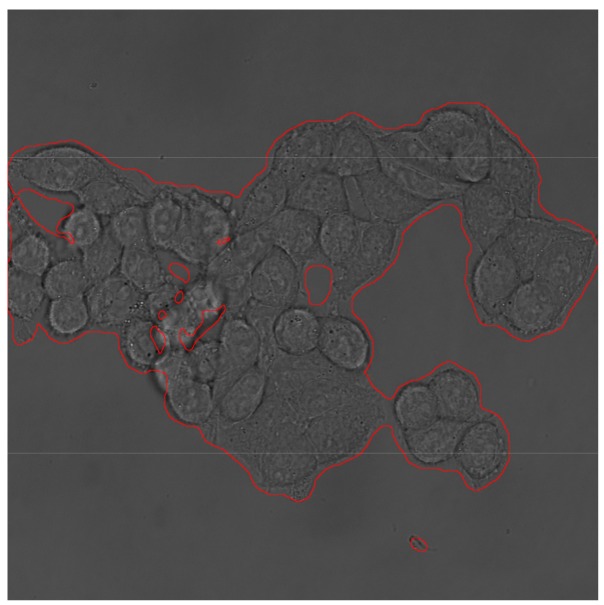
Original brightfield image outlined with segmented area.

1. Open brightfield image and equalize its histogram, set the portion of saturated pixels to 0% (Figure 1).
run(“Enhance Contrast...”, “saturated=0 equalize”);
2. Perform Canny edge detection with Gaussian kernel radius pixel resolution set according to Table 1, Low threshold set to 0.1 and High threshold set to 8.0.(Figure 2).
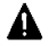
**CRITICAL STEP** Especially high threshold setting has a significant impact on the final segmentation, and should be first to consider when adjusting the procedure for optimal result.
run(“Canny Edge Detector”, “gaussian=1.75 low=0.1 high=8”);
3. Run Maximum filter with radius set to value according to Table 1. This filter creates a running window that replaces the central pixel with the maximum value of the neighboring pixels. In the edged image, this filter fills most of the intracellular space (Figure 3).
run(“Maximum...”, “radius=5”);
4. Perform morphological operation Closing with number of iterations set to 10 and count set to 3. This fills the remaining small holes in the image.
run(“Options...”, “iterations=10 count=3 pad do=Close”);
5. Run morphological Opening with number of iterations set to 10 and count se to 3. This eliminates small structures (debris, etc.) localized outside the cells.
run(“Options...”, “iterations = 20 count=3 do=Open”);
6. OPTIONAL STEP Run morphological Erode with iterations set to 2 and count set to 3. This step reduces the effect of morphological dilation introduced in step 3 by Maximum filter (Figure 4).
run(“Options...”, “iterations=2 count=3 pad do=Erode”);
7. Overlay the outline of segmented areas with the original brightfield image and check the precision of segmentation. If necessary, adjust the parameters of procedure, focus especially on High threshold setting of Canny edge detection (Figure 5).

## 4. Expected Results and Discussion

**Figure 6:**
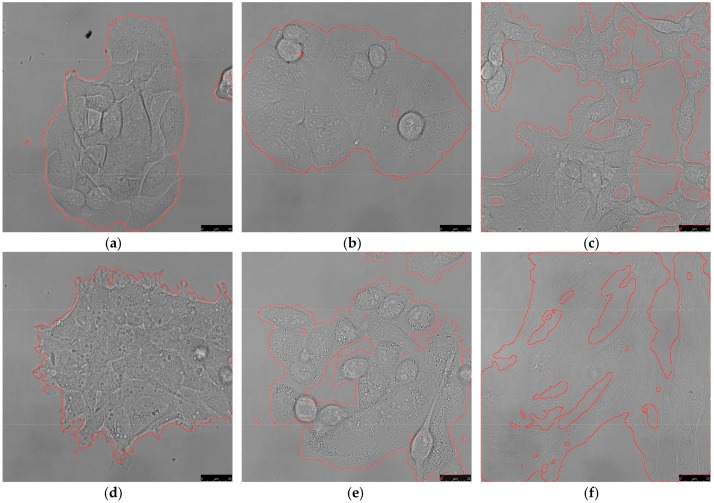
Results of brightfield segmentation using the same process parameters on various cell types. Except for the added micron scale, the images were not adjusted in any way before segmentation. (**a**) HaCaT; (**b**) MCF7; (**c**) 3T3 murine fibroblasts; (**d**) HEK-293; (**e**) MDA-MB 231; (**f**) normal human dermal fibroblasts (NHDF). Scale bar 25 µm.

The procedure can be performed on various cell types and for images acquired with the same optical setup, and it does not need any further adjustments in most cases. Figure 6 shows the results of segmentation on six cell lines with various morphologies, acquired with a 63× water objective (pixel resolution 110 nm/pixel) and using the same parameters in all cases. Since the procedure is based on edge detection, it works best on cell types with clearly visible borders (typically HCT-116, MCF7, HT-29). It is possible that cells with not-so-distinctive borders, such as fibroblasts, may need adjustment of procedure parameters for better separation of intracellular space from background. In such a case, a High threshold parameter of Canny edge detection should be first to be modified. Since the rest of the procedure is based on morphological operations, changing the parameters of edge detection may require further adjustments of other steps as well. For example, as is shown in Figure 2, setting High threshold too high can, on the one hand, reduce the probability of detecting noise but, on the other, detect a lesser amount of edges inside the cells that would need different parameters of Closing.

**Figure 7:**
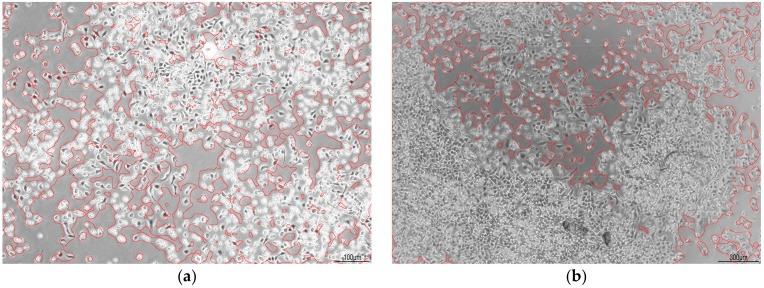

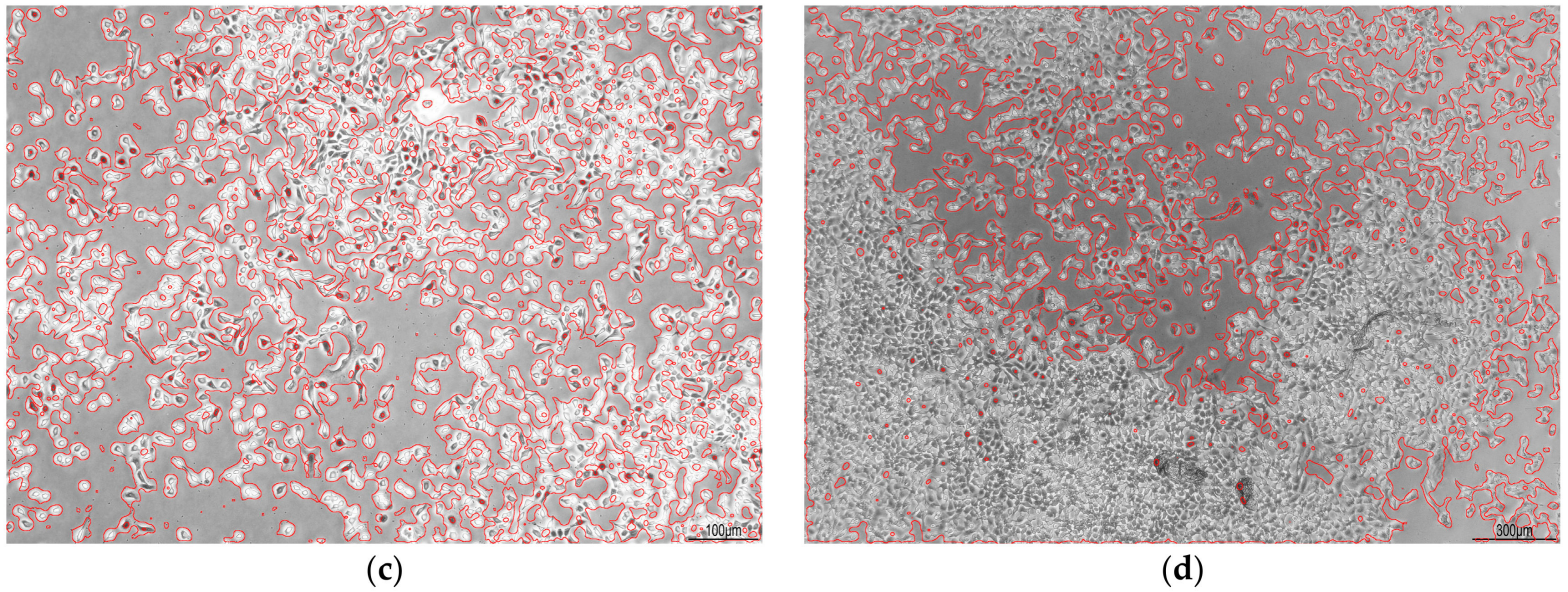
Images of segmented NHDF acquired with 5× objective with varying confluency and digital zoom. In (**a**,**b**) without changing parameters of morphological operations, in (**c**,**d**) with number of iterations of Closing and Opening set to 2 and 10, respectively. Scale bar in (**a**,**c**) 100 µm, in (**b**,**d**) 300 µm.

Analysis of images acquired with less powerful objectives (<20×) requires changes in procedure parameters with respect to the different area (in numbers of pixels) occupied by a single cell. Figure 7 shows pictures of normal human dermal fibroblasts (NHDF) acquired with 5× objective (pixel resolution >360 nm/pixel). Parameters were adjusted according to Table 1, and High threshold setting in the Canny detection step was set to 9. In Figure 7a,b all the remaining parameters were kept at default values from Section 3. As can be seen in the pictures, the process is a little too coarse to precisely copy the shape of cells. This can be fine-tuned by adjusting parameters of morphological operations to make the procedure more accurate. In Figure 7c,d are the same pictures processed with number of iterations of Closing set to 2, and number of iterations of Opening set to 10.

**Figure 8:**
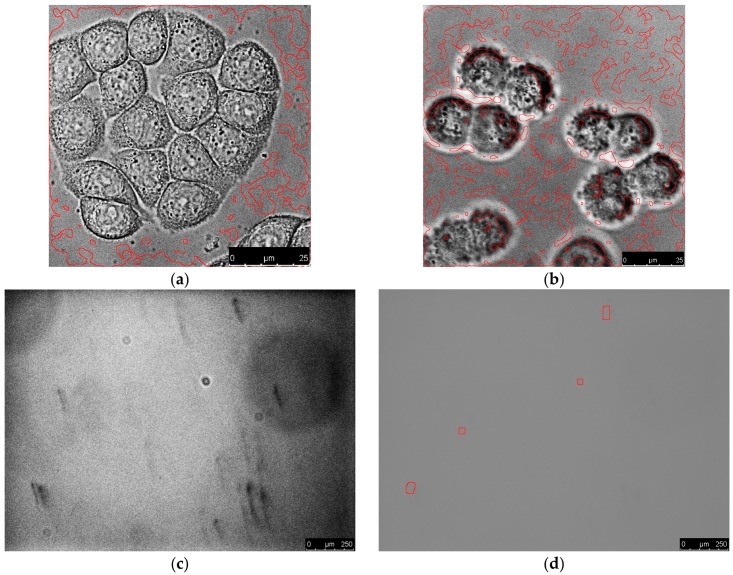

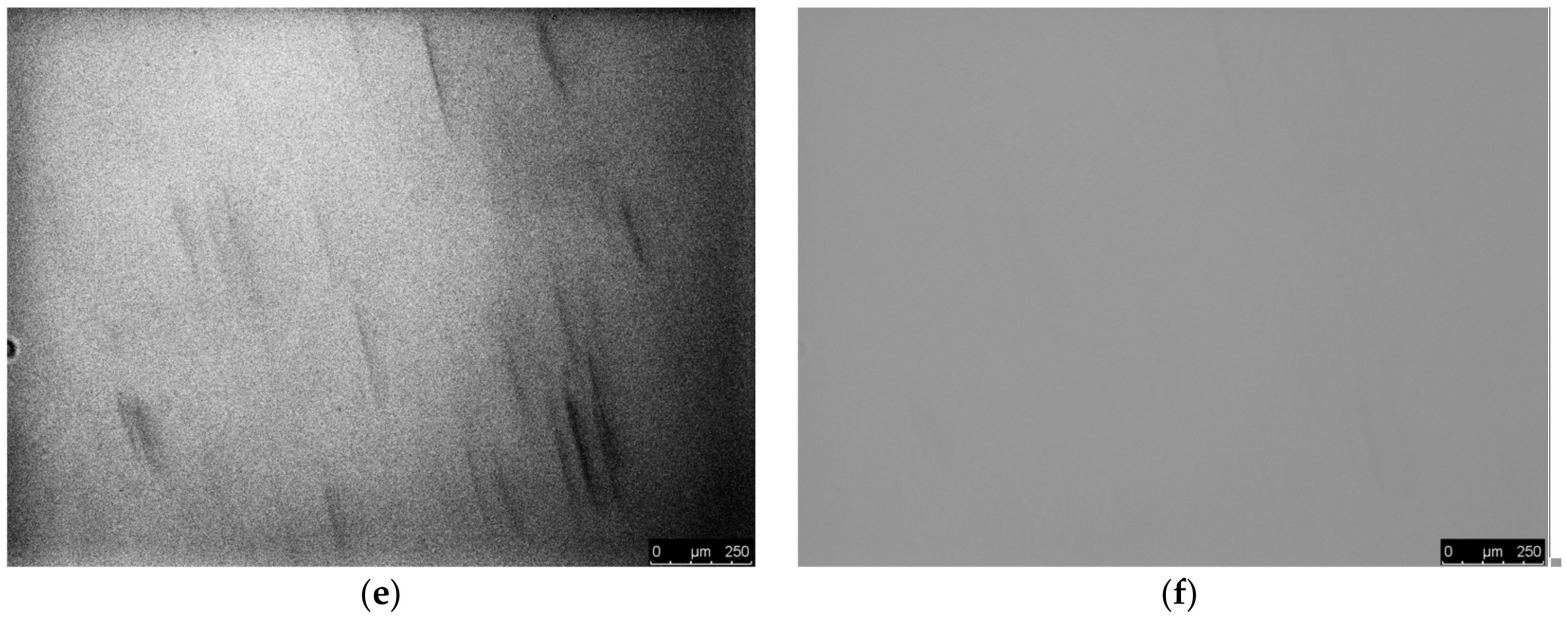
(**a**,**b**) Results of processing insufficiently illuminated images. Images are shown after histogram equalization. Scale bars 25 µm.(**c**–**f**) Blank fields of view with uneven illumination and impurities. (**d**,**f**) Original images with highlighted areas that were positively detected; (**c**,**e**) Images after histogram equalization. The process parameters were the same as described in Section 3 in all cases. Scale bars 250 µm.

The procedure requires images acquired with a properly set-up microscope, and with decent contrast. Examples of poor-quality images and blank images processed with the parameters from Section 3 are shown in Figure 8. Figure 8a,b were acquired with insufficient illumination (with (b) being slightly out of focus) which leads, after histogram equalization, to strongly pronounced noise that is incorrectly recognized as a cellular structure. Figure 8c,d show an empty field of view after equalization and original image with highlighted areas that were positively detected as cells. Although the original image seems to be a uniform background, equalization reveals impurities that possess a certain level of contrast. As the procedure is simply based on an edge detection and morphological operations, it is no surprise that these areas are highlighted. However, as can be seen in Figure 8e,f images with less contrast inequalities in illumination are processed without any false positive detection.

Precision of segmentation was validated against manual segmentation performed by two biologists on the set of 10 images of various sizes and cell types. The parameter of interest was the size of area positively marked as being occupied by cells. For automated segmentation, the process parameters were adjusted according to Section 3, to match the properties of images. The results, represented as a mean of differences between automated and manual segmentation (in % with the result of manual segmentation considered to be 100%), are summarized in Table 2.

The automated segmentation turned out to be more lenient than manual, resulting in 7.9% (±4.8%) and 8.5% (±6.2%) positive difference. There are few reasons why the area detected by the process is, in general, larger. The main reason is impurities, such as cellular debris, etc., that would not be marked by a biologist as a cell, but can be detected if they contrast and are big enough. Another reason is a presence of complicated cell protrusions that might be either omitted by manual segmentation, or fused together by the automated process, with both ways resulting in a larger area detected by the process.

## Figures and Tables

**Table 1:** Recommended values for pixel resolution dependent filters.

Pixel Resolution (nm/pixel)	Gaussian Kernel Radius	Maximum Filter Radius
90–180	1.75	5
180–270	1.5	2.5
270–360	1.25	1.67
360+	1	1.25

**Table 2:** Quantitative evaluation of precision of automated segmentation against manual segmentation.

	Number of Analyzed Images	Mean of Differences between Automated and Manual Segmentation ± SD
Biologist 1	10	+7.9% ± 4.8%
Biologist 2	10	+8.5% ± 6.2%
